# Population-Sequencing as a Biomarker for Sample Characterization

**DOI:** 10.1155/2013/861823

**Published:** 2013-12-08

**Authors:** John P. Jakupciak

**Affiliations:** Cipher Systems, 2661 Riva Road, Annapolis, MD 21401, USA

## Abstract

Sequencing is accepted as the “gold” standard for genetic analysis and continues to be used as a validation and reference tool. The idea of using sequence analysis directly for sample characterization has been met with skepticism. However, herein, utility of direct use of sequencing to identify multiple genomes present in samples is presented and reviewed. All samples and “pure” isolates are populations of genomes. Population-Sequencing is the use of probabilistic matching tools in combination with large volumes of sequence information to identify genomes present, based on DNA analysis across entire genomes to determine genome assignments, to calculate confidence scores of major and minor genome content. Accurate genome identification from mixtures without culture purification steps can achieve phylogenetic classification by direct analysis of millions of DNA fragments. Genome sequencing data of mixtures can function as biomarkers for use to interrogate genetic content of samples and to establish a sample profile, inclusive of major and minor genome components, drill down to identify rare SNP and mutation events, compare relatedness of genetic content between samples, profile-to-profile, and provide a probabilistic or statistical scoring confidence for sample characterization and attribution. The application of Population-Sequencing will facilitate sample characterization and genome identification strategies.

## 1. Background

A paradigm shift in microbial-forensics is emerging due to the potential of interrogating comprehensive genome content of samples via direct application of Next-Generation sequencing. The innovative process of sequencing populations of entire pathogenic mixtures as opposed to sequencing isolates, from diverse samples, and comparing to a standard reference is described. The approach greatly accelerates and vastly shortens the interval from time/point of contact, to specific pathogen and strain identification. This abbreviated timeline is critical to respond to and treat exposure victims. Moreover, tracing the origins of strains is accentuated, which facilitates rapid investigation. Population-Sequencing will be more productive to provide genome information than to singly clone one genome from the sample population and then analyze that genome individually cultured from it. Herein, direct and practical solutions as well as some current limiting dogmas in this area are presented.

**Figure 1:**
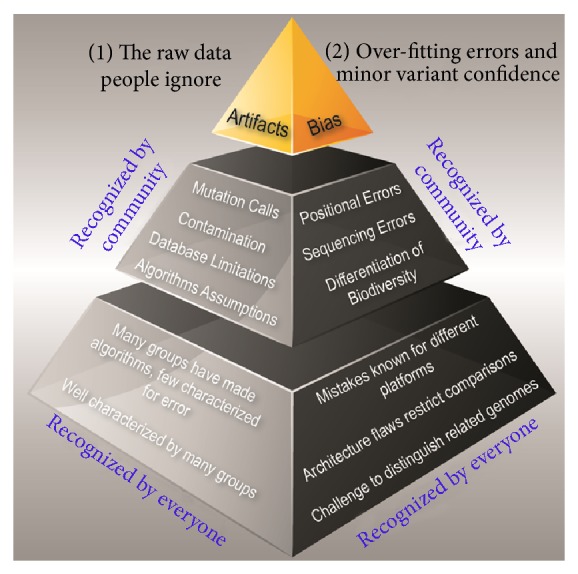
Metagenomic sequencing has its own set of limitations including system bias and random noise. The general science community is aware of many of the limitations. A subset of challenges to accurate genome identification is recognized by those active in sequencing research, while two factors (artifacts and bias) are rarely adequately resolved.

Whole genome sequencing (WGS) analysis has become feasible for molecular biology and comparative genetics due in large part to available tools, such as BLAST [1], and Mummer [2]. However, existing tools have recognized limitations and require refinement for forensics applications to address data artifacts and pipeline errors, including sequencing errors and algorithm bias (Figure 1). If artifacts, errors, and limitations are not handled appropriately, no amount of computing will derive accurate answers [3, 4]. Bioforensics requires new methods and tools to reconcile errors, determine relationships between samples, and distinguish between real signal versus noise, to accurately identify organisms present in samples and provide confidence identifying major/minor populations and their concentrations [5, 6]. A process for Accurate Bioinformatics Calibration (ABC) is needed to address errors recognized by the community and to resolve bias and artifacts. In addition, sequencing instruments were designed to facilitate genome assembly and not intended for population analysis. Comprehensive detection of microorganisms in samples with accurate identification at the strain level is a challenge in microbiology, particularly when complex indigenous communities or subpopulations varying in viability, activity, and physiological state are investigated [7]. Accurate characterization of a sample is dependent on ascertainment of the genetic variation between microorganisms with resolution down to strain level. Development of bioinformatics tools to process volumes of sequence data provides solutions to many challenges, including bacterial forensics where Population-Sequencing identifies genome content of samples and differentiates between samples by creating genetic diversity profiles unique to each sample.

Whole genome analysis: genomic analysis progressed through comparisons of sequence fragments to genes to whole genomes [8, 9]. The benefit of whole genome analysis over a selected panel of signature markers was made evident in human forensics and analysis of mitochondrial DNA [10, 11]. These pioneering applications of analysis of entire genomes used sequence information to measure dynamics of entire sequences of interest. These examples point us in the direction of looking at comprehensive sequence information to characterize samples, rather than analysis of discreet regions of genomes, intended to describe one genome in the mixture: the product of consensus sequencing. DNA analysis of human mitochondria (huMT) showed the resolution of whole genome analysis for genome identification and differentiation, which can be applied to microbes for adoption of whole genome analysis for identification of individual genomes from metagenomic mixtures [12]. Instead of classical methods and instead of DNA methods that consider small percentages of the genome (PCR, etc.), whole genome sequencing can be directly applied to microbe identification [13–15].

## 2. Biodiversity Improves the Accuracy of Population-Sequencing

A wide range of molecular detection technologies are being developed and used for genomic analysis of pathogens and their communities [8, 16]. Increasingly it is understood that pathogens from the environment show variability in sequence and that any given species contains strains that are related but not of identical reference sequence [17]. Only recently are microbial communities beginning to be characterized as metagenomic samples for the purpose of community profile-to-profile comparisons [18]. Based on fundamental knowledge of the pan- and core-genomes and high content/deep sequencing capabilities of sequencing platforms, it is possible to characterize genetic variation within and between bacterial pathogens, multiple isolates, and commensal near neighbor species and establish a profile of the major, and to some extent, minor genetic content of samples [19]. Metagenomic approaches based on direct sequencing represent powerful tools to compare genome profile of mixtures [20–23]. Alternatively, microbial diversity in metagenomic samples has been assessed by analysis of conserved marker genes, for example, 16S rRNA genes [24]. Initial sample analysis investigated the variety of genes in samples [25]. However, comprehensive descriptions of genomes and accurate identification at the strain level of genomes present and statistical measurement about the confidence on concentrations or relative abundance are needed. The resultant stain-level profiles are analogous to less sensitive profiles generated from microbiome studies, which assign bacteria into genera, often precluding resolution even to the genus level [17, 26, 27].

Direct sequencing of populations provides a more comprehensive analysis of genetic content within the sample [28, 29]. However, evaluation of sequence data output requires appropriate interpretation without over-fitting (Figure 1). One way to improve accuracy is to develop Bayesian approaches to compare data between pathogens and their near neighbors. The models resolve genetic mixtures to the maximum, information theoretic, justifiable extent to calculate statistical confidences assigned to both genome identity and sample population proportions. Still caution when interpreting sequence raw data is required because there are also many types of system error and artifacts identified when sequencing populations of lineages of biothreat agents. While some system errors can be observed when using sequencing standards, instead of sequencing standards, what must be developed are powerful probabilistic methods that scour data sets to filter-out artifacts, enabling a probable estimate of accurate assignment of sequence data to genomes: distinguishing between artifact and sequence data useful for accurate genome identification. Horizontal gene transfer events and similar variants may arise repeatedly given an adequate supply of exogenous DNA, an environmental DNA pool, and positive selection pressures in the environment that maintain that variant. This integration of exogenous DNA may allow optimization of highly adaptable regions of the genome, thus enhancing the fitness of the genome for a particular niche similar to the superintegron region [30]. Indeed, enhanced characterization can be exploited by using incorporating approaches to identify genomic adaptive signatures of natural selection [31].

**Figure 2:**
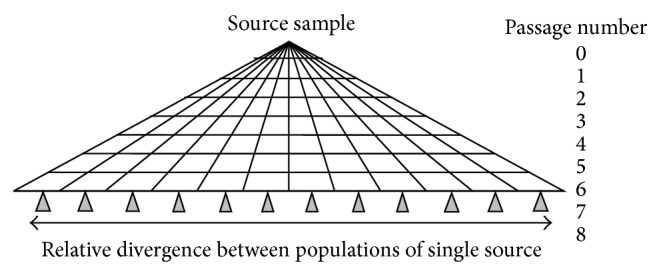
Bacterial strains from a single colony are divided into 12 parallel cultures before passaging occurs in different media conditions, eight consecutive times. At the end of the experiment, clones from each “lineage” are selected, sequenced, and analyzed for genetic drift and relatedness.

To advance metagenomics, new insights on system errors and artifacts needed to be understood and solutions incorporated into bioinformatics approaches. There are efforts to reconcile sample analysis performed at different levels, individual genes to population characterization [32]. This is especially true in the case of organisms modified by environmental pressures or selective pressures of host or manipulation in laboratories. Under the Bio-agent Early Warning Detector program (BEWD) (Bio-agent Early Warning Detector (BEWD) was a DTRA initiative to evaluate capability of Population-Sequencing for sample characterization without sample prep or culture, involving sequencing of extracted DNAs from mixtures and subsequence probabilistic matching of sequence information to describe the populations of genomes present in samples from water, soil, air, and clinical specimens) population-sequencing was applied to characterize the extent of modification and variation of biothreat agents cultured in the laboratory and measured, to a limited degree, expectations on such comparison and their difference from variation by different natural environments. When sequencing is used to assess the variation, few bioinformatics tools, reference free, and reference dependent bioinformatics strategies are robust enough to account for genetic diversity in samples and system errors and artifacts. Sequence variants must be nonarbitrarily confirmed in both forward and reverse reads at a rate above the background noise level of sequencer machine error. A similarity distance metric determines if compared genomes fall within a range of near relationship. Using sequence data from strains of *Bacillus anthracis* and other biothreat agents, comparisons resulted in successfully attributing known related strains together, and excluded near relation of known unrelated strains (Figure 2).

The major strengths of these forensic methods are the nonarbitrary determinations of data validation and relatedness metrics, as well as the ability to compare microbial genomes with or without a reference database of related genomes and addresses prior assumed challenges; for example, the definitions of “pure” and “isolate” are truly more appropriately described as populations of genome diversity. While there are many computer programs for sequence analysis, BEWD overcame prior perceived limitations, for example, the novelty of how sequence information is divided into fragments of sequence data for analysis. Variability across sequencing runs was properly addressed. Prior methods used a set *k*-mer size, but computational demands limit efficiency and accuracy of such approaches [33, 34]. Analysis of population sequencing data revealed that the fraction of the total probes that provided experimental results consistent with the predicted results decreased substantially with increasing divergence of the tester strain from the reference strain. Comparison of strains across the phylogenetic tree produced predictable results; that sequences of targeted genomes compared to a reference that is distant from the target receive a low probability score and targets compared to references that are highly related to the target receive the highest probability score.

Traditional pathogen detection and characterization approaches limit sample comparisons to matching clonal isolates against reference libraries to first identify the species and/or strain of the microbe [35, 36]. After the best matching reference genome is found, variations from the reference genome sequences are discovered. Data sets possessing similar sequence variations are clustered together by phylogenetic analysis. Such approaches have bias on initial selection of references. The chosen reference material is assumed to be the representative genome out of the entire population. Instead, the genetic content of the entire population should guide selection of genomes for comparison. This avoids arbitrary reference selection and results in comprehensive genetic analysis of samples.

Microbial forensics needs a paradigm shift away from the clonal isolate versus reference genome approach. Foremost, real world samples rarely exist as homogeneous populations of identical clones. Even populations that derive from single cells acquire mutations that are not equally shared by all members. Archived cultures contain variations of genome content and those variants occur significantly even in theoretically clonal populations of slowly evolving bacteria. While genome scale synteny analysis of relative gene-order conservation between species can provide epidemiology and evolutionary dynamics, forensics requires further information to determine how one sample is associated with one source. These “metagenomic” population samples require bioinformatics approaches that deviate away from the clonal isolate paradigm in order to accommodate potentially hundreds of distinct genomes that may or may not be part of any database.

## 3. Introduction to Population-Sequencing 

Although nucleotide and aminoacid sequence-based approaches have been used in the past for inferring microbial evolutionary relationships, over the last 10 years, these methods have been increasingly used for typing and characterizing their populations [37–39]. New awareness of the vulnerability of national infrastructure to the intentional introduction of pathogens or pests has led to the enhancement of programs for prevention and preparedness [40]. A necessary component of a balanced biosecurity plan is the capability to determine whether an outbreak may have been deliberate, to trace that outbreak to its source and to identify those responsible for it. Sequencing methods provide standardized and unambiguous data that are portable through web-based databases with direct access to the information needed to identify and monitor emerging pathogenic agents [41, 42]. More importantly, sequence data, unlike many other forms of molecular typing data, provide direct genealogical information that can be used efficiently to estimate phylogenetic relationships and parameters associated with population dynamics [43]. Biodefense preparedness begins with interpretation of the appropriate data to detect, respond to bioagents, prevent an outbreak, and attribute a threat.

Microbial forensics is an emerging discipline that blends elements of numerous disciplines including microbiology, forensic science, and agricultural sciences. Sequence mapping capabilities which attempt to forensically characterize microbiological evidence in support of attribution are limited to detection of pathogens, often in a preselected, not blinded approach. Forensics analysis requires more than detection of single genomes in the population mixture, but a detailed characterization and comparative assessment of the sample genetic contents. Superficial detection approaches have left gaps in forensic science; fortunately, analysis of populations from single sources has developed microbial characterization tools that fill the knowledge and capability gaps.

With the advent of next-generation sequencing approaches allowing for metagenomic sampling of microbes from outbreak scenes [44], data are available to both characterize novel genomic elements of pathogens and trace those novel elements through evolutionary history for both identification and tracking purposes. Because these data are relatively new and constantly evolving, in terms of read lengths, formats, quality, innovative and flexible pipelines for data quality control checks, genome assembly, SNP detection, visualization for comparative genomics, genome annotation, ortholog assignment, and phylogenetic analysis that account for artifacts and bias were developed.

### 3.1. The Advantage of Population-Sequencing

Population-Sequencing establishes the capability to rapidly detect all especially dangerous pathogens (EDP) and biothreat agents, emerging or engineered, through analysis of sequencing information from one run. Commercial R&D is focused on personalized medicine applications and not on timeliness. Technology advances, suitable for modification and adaption to bioagent population detection, have emerged more rapid than could be anticipated and planned for in a traditional five-year POM cycle. In fact, cost of sequencing has continued to decrease, but the cost associated with handling and processing large volumes of sequence data has increased dramatically [45–48]. Bioinformatics tools are now needed to solve two challenges: namely, the challenge of accurate genome identification of metagenomic samples and reduction in the burden of sequence data analyses.

Direct-Sequencing of populations is an improvement for bacterial forensics. On account of diversity of several genomes and their variations in samples, probabilistic matching of many-to-many is greater than one-to-one; it is possible to compare genetic population contents of evidence to populations of source. The comprehensive genetic contents of a sample represent the plurality of biomarkers for identification, even as they evolve, dependent on the diversity of the sample's starting population; hence only one source will have the same comprehensive diversity and thus match evidence to single source. Understanding the direction of that diversity is essential to make comparisons of samples with different genetic content.

### 3.2. Experimental Design

1. Observe DNA sequence mutations arising from an originally clonal isolate of biothreat material, for example, *Bacillus anthracis* strain Ames, when cultured under stressful conditions in the laboratory.
2. Determine the strengths and limitations of whole genome sequence analysis for characterizing variation between similar substrains.
3. Advance methods for determining “relatedness” between microbial samples.

A single colony of biothreat material, for example, *B. anthracis* strain Ames (BEI#NR-411), was passaged into 12 different plates. These twelve bacterial cultures were maintained separately over the course of seven more passages. Each culture passage was started with a single clonal colony streaked out on a petri dish. This created a single genome bottleneck at each passage step. Mutational variations differentiating each lineage were thus a result of initial variation in the source clonally derived culture plus mutations accumulated during the course of the eight growth and passage steps. A schematic diagram of the experimental design is shown in Figure 2. DNA was subjected to Illumina GA II sequencing and 2460 Mbp reads for community DNA samples.

**Figure 3:**
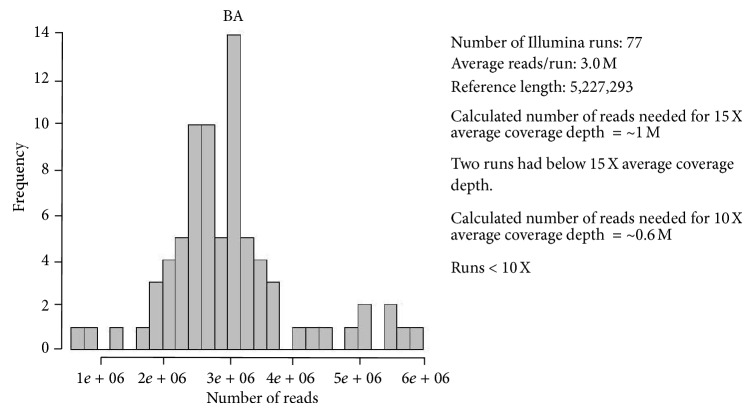
Plots of sequencing reads from *B. anthracis*.

A summary of the raw single ended read data for the *Bacillus anthracis* samples (Figure 3).

Testing bioinformatics pipelines for sequence assembly and analysis of bacterial genomes was done to evaluate performance of different assembly software selected, based on popularity and third-party performance comparisons. All of these methods can perform gapped and ungapped alignments against a reference genome and manage short (<200 bp) and long reads (>200 bp).

### 3.3. General Nucleotide Diversity

**Figure 4:**
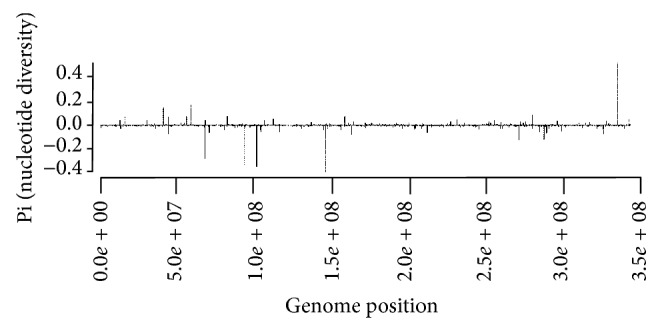
Example of subpopulation genetic diversity of passaged strains measured against progenitors.

Comparison of major genomic features of *B. anthracis* (Figure 4). The SNPs and lack of SNPs are based on genome assembly and alignment of the clone from the final passage compared to the reference isolate genome. The isolate was initially divided into 12 independent lineages that were separately, but in parallel passage, exposed to the same bottle necks, processed by the same selective pressure, and extracted and sequenced on an Illumina GIIA. Each nucleotide variant present in the passaged material which is visualized has a peak above. Each nucleotide variant absent is represented as a decrease in signal.

The results show that the passaged genome is one component of the population of genomes from the isolate. Although the initial starting material was characterized by consensus sequencing to be *B. anthracis Ames*, there are many variants of the Ames strain present in the initial population. This comparison is one of 80 possible variants that represent major and minor components of the population. All the positions and types of variants were catalogued and used to build SNP phylogenies.

### 3.4. Nonarbitrary Genetic Detection

Identification of genomes in mixtures is dependent on correct assessment of sequence data and system errors that manifest in the sequence data output. For example, the use of Illumina data means that millions of reads are available to build statistical confidence of identification but at the same time generates millions of reads that do not correspond to reference data. This noise is not novel emerging genome sequence data. The artifacts were verified through repeatability assays where the same biothreat agent is measured 20–70 times to determine useable sequence data.

### 3.5. Analysis of Sequence Gaps

**Figure 5:**
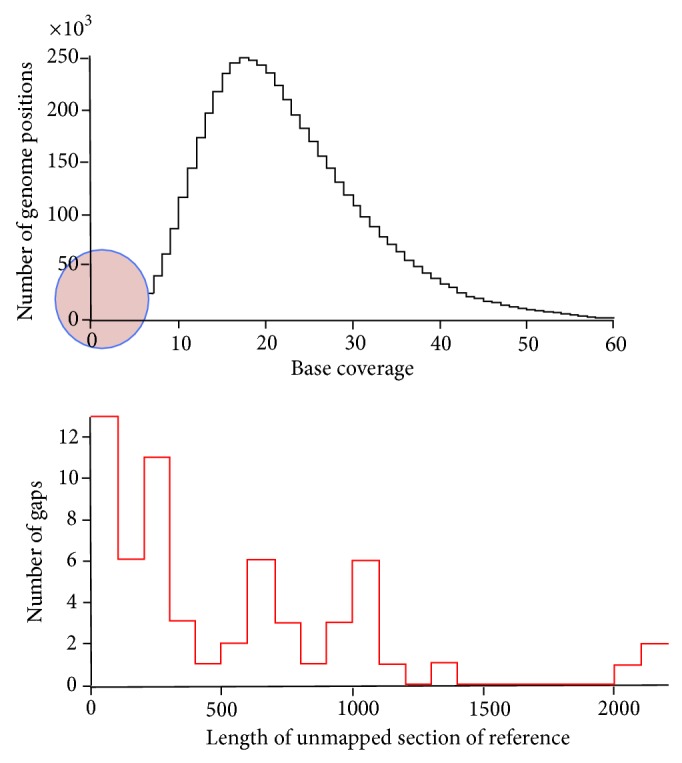
It compares read *n*-mers to reference genome *n*-mers and counts the number of hits per genome *n*-mer (signal) and misses (noise). The separation between signal and noise varies with read depth. The detection threshold that maximizes signal *n*-mer retention and noise *n*-mer rejection is to be used, and will vary with number of total reads (coverage).

Analysis of sequence data on each isolate computed the position and coverage across the genome.  Figure 5 shows a spike at zero which indicates that there are many, potentially millions, sequence reads without matching fragments. These reads represent a number of challenges for direct sequence analysis. The distribution of sizes in gaps is plotted in Figure 5(b). Such data if used for a single target and not for comprehensive analysis of populations which build the statistical confidence to derive matches of materials to source, enabling attribution, is not unexpected but illustrates the required probability of performing multiple matches of the genomes in the mixture of populations for adequate characterization of sample genetic content.

**Figure 6:**
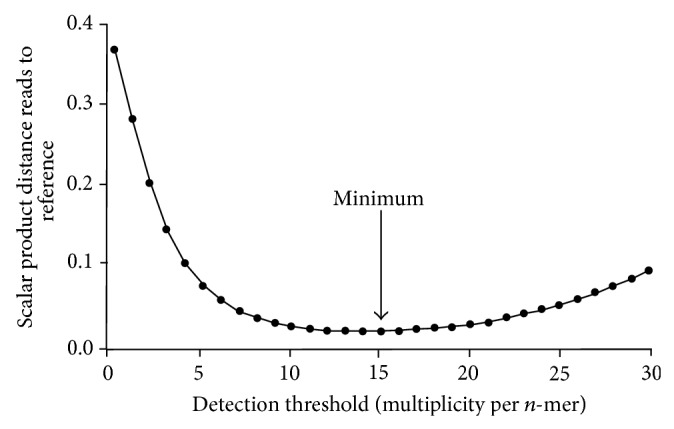
Shows the plot to determine the minimum or optimized output. As the threshold is moved to more stringent criteria there is a loss of signal as well as loss of background, so shown by the increasing tail of the plot. Hence the reference selection is critical for accurate identification.

Artifacts in sequence data do not follow the trend of the signal, rather the noise continues to grow; hence the potential for inappropriate use of such data as meaningful sequence information resulting in errors increases. As the sequence coverage increases the confidence in accurate matching approaches a limit. However, the noise grows linearly. These generate the signature of the genome, which is the list of all *n*-mers in a genome. As the noise increases, there is bleed-in of strings with high confidence of matching. Figure 6 plots the signal to noise ratios.

Understanding rare variants is a challenge to metagenomic analysis [49, 50]. A calibrant is needed [51]. The threshold can be calculated for every sequence run and for each genome. This calibrant can be universally applied for optimization of the *n*-mer count threshold. For a selected sequencing depth, a calculated threshold observation multiplicity for each *n*-mer can be determined with the calibrant (Figure 6).

### 3.6. Bacterial Forensics Calibrant

The ABC calibrant determines the multiplicity. The measurement is based on comparison of the sequence vectors. As coverage is built on the fragment, edges of the string vectors are compared. Strings that map to the wrong site generate noise along the edge. The BFAST algorithm compared reads and performed alignment. SAMToolsMPileUp feature overlapped reads at their reference genome alignment locations for the purpose of detecting consensus or variation. Custom processing scripts searched gaps, specific mutation calls, and other criteria to assess the continuum of sequence data against the dataset threshold.

Bioforensics is dependent on matching sequence data to references. Probability of obtaining matches above the threshold of established criteria need to be calculated for each matched position to a reference. Databases of genomes are also not necessarily needed, because the comprehensive genetic content of samples and their genome profiles can be compared to other sample profiles. The limit is that not all sequence data is compared because of artifacts and error, but genome identification via direct analysis of appropriate sequence data establishes the profile and profiles can be compared. Sequence information is triaged to determine the high quality data for comparisons and identification of known genomes and for clustering of genomes outside of reference databases.

### 3.7. Detect Constituent Organisms in Sample

For rapid read mapping and read assignments against database entries, a precalculated *n*-mer database is needed. A tiered database architecture has the potential to respond fast because of locality of data with configured match queries routed through the database. This is accomplished in part by a hybrid direct memory address array and binary tree. There is a tradeoff between memory size and the number of comparisons. A large *n*-mer value requires too much memory and a small *n*-mer value creates loss in match specificity.

Matching is defined as vector space where the sequence content is compared as a motif against the sequence space of references. Comparisons can also be made between samples in the absence of reference(s). Comparisons to determine the genome content and comparisons to determine if samples are related or linked by common ancestry, for example, attribution, the vectors of sequence data are evaluated.

In general there would be 16 million possible combinations if the vector size was constrained to a 12-mer (4^12^). All the vectors are compiled to build an index where each vector element corresponds to a unique *n*-mer. The value of each element is 1, if that element is present in the genome (forward or reverse) and zero otherwise. This processing is applied to the collection of assigned sequence reads. Vector space is a composite of 1 and 0 for reads and the matching genome compared by a dot product for distances, the direction of the cosine.

Determining the vector matches and establishing the threshold via the use of the calibrant enables comparison of sequence data for identifying sample with source. Probabilistic approaches use Bayesian likelihoods to consider two important factors to reach an accurate conclusion: (i) *P*(*t*_*i*_/*R*) is the probability that an organism exhibiting test pattern *R* belongs to taxon *t*_*i*_, and (ii) *P*(*R*/*t*_*i*_) is the probability that members of taxon *t*_*i*_ will exhibit test pattern *R*. The minimal pattern within a sliding window will assist investigators on “whether” and “how” genomes vary.

The probability calculation procedure is based on the average relative position and frequency of lineage terms. More weight is given to broader, more general terms occurring at the beginning of a lineage (e.g., kingdom, phylum, and class), and less weight to narrower, more detailed terms that occur at the end (e.g., family, genus, and species). To compensate for the fact that some lineages contain more intermediate terms than others (e.g., including super- and/or subclasses, orders, or families), the calculation normalizes for total number of terms and weights each term according to its average position among all lineages tested, rather than an absolute taxonometric rank. The end result is a very fast, computationally simple technique to assign higher probability scores to lineages that occur more frequently and lower scores to lineages that occur only rarely. Groups of phylogenetically related organisms receive similar lineage probability scores, even if actual matches to the query genome are unevenly distributed among individual members of the group.

## 4. Error Is More Than Traditional View of Sequencing Error: Traditional View

Due to inherency of base calling errors in all DNA sequencing methods, trusting all sequence variants that appear in raw sequence data is not recommended. Even when data comes from low error rate platforms under best conditions, error rates of at least 0.1% will create false SNPs on the average of once every 1,000 bases [52, 53]. Over the course of a 5 Mb genome, which adds up to a total of 5,000 false SNPs multiplied by the average coverage depth of the genome. Even worse, a 0.1% error rate is much lower than what is expected in most sequence data sets at the moment.

### 4.1. Application to Bacterial Forensics

**Figure 7:**
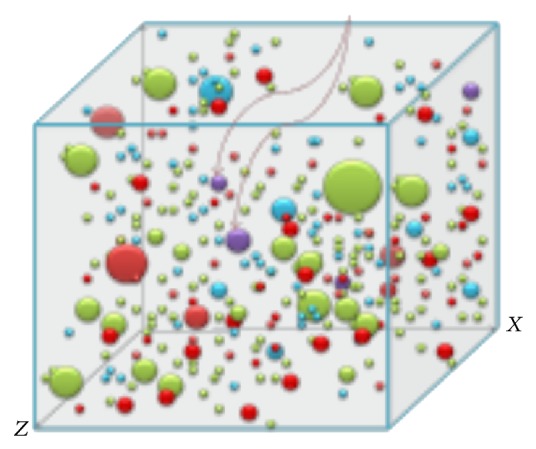
Difference genetic subpopulations. Red arrows indicate related genomes. The entire population's genetic content can be used to match and compare samples.

While millions of sequence reads can be generated, select reads function as biomarkers to provide useful information for accurate characterization populations in samples (Figure 7). Analysis of comprehensive sample content also provides insights about sample context—the current population at large and how does that sample or genome fit into the population and can that information give any merit or accuracy about the answer.

The population data can be used to calculate the degree of relatedness and how close of a match the samlples are and if the interpretation aligns with the evidence or a false direction. Measuring the direction and magnitude of genetic variation facilitates distinguishing between environment influence on the populations and lab manipulation: potentially indicating if an outbreak is natural or intentional. Population sequencing not only identifies specific genomes, it provides a confidence indicator helpful for decision-making about how it is related to others and within the population.

### 4.2. Microbial Forensics

**Figure 8:**
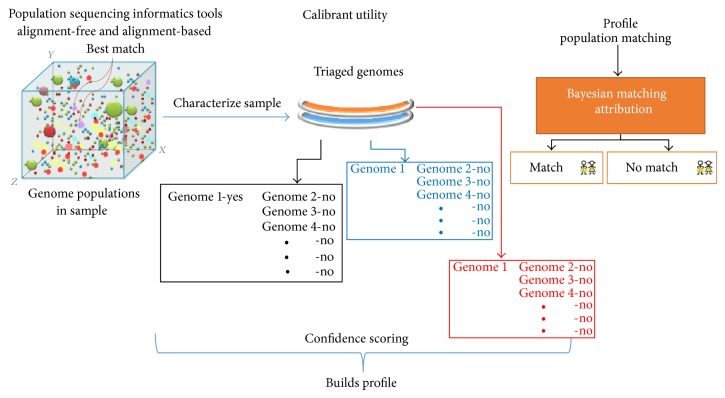
Tiered population characterization. Metagenomic sequence data is directly analyzed with novel informatics tools, genomes scored for statistical confidence, and profiles compared for attribution. Each genome is treated differently from the next.

The main purpose of microbial forensics is to determine whether or not to attribute a microbial pathogen sample back to a suspected source [54, 55]. Interpreting diversity of complex mixtures and subtle variations in isolate populations represents a critical need for bioforensics. Sample characterization and traceability of genetic contents back to source are dependent on genome identification of specific targets within the sample, comprehensive analysis of the mixture of populations present, detection of major and minor variations in the identified genomes, and comparison of the sample genetic profile against other sample profiles (Figure 8). Metagenomics is an emerging discipline for microbial population(s) analysis based on sequence information obtained directly from samples without culture purification. “Population-Sequencing” establishes comprehensive descriptions of genetic content of samples and creates a profile of that genetic content specific for each sample.

The profile, unique to each sample, represents specific target organisms, for example, biothreat agents, as well as auxiliary genomes, for example, contaminants, related near neighbors, or commensal organisms indicative of certain factors either from their environment or the fashion of sample handling or manipulation. On account of diversity, the population, or cloud, of genetic material is unique to each sample. While specific targets or core genomes might be in common between samples, the pan-genome or cloud of variation will be unique dependent on the breadth of genetic diversity of the starting material and selective pressures. Sequencing the cloud of diversity establishes accurate sample-to-sample comparisons.

A wide variety of tools have been developed, but all methods, except for those that take into account the system errors in the entire pipeline of sequencing, are flawed and generate inaccurate genome identification. With appropriate delimiters set across sequence information, reorganized databases to enable accurate searching, and alignment-free coupled with alignment methods to enable accurate matching.

The sequence data obtained from the device will not only include sequences from biothreat agents, but also other “background” sequences from hosts and natural microbial community, or both. The database used for the comparative purpose should cover as much as information to take advantage of current advancement in genomics. The calibrant is part of a bidirectional process to compare database entries and sequence sample data sets. The calibrant is establish by calculating the distance between the database entries and the same genus, species, or strain designation and the distances for the assigned reads are also calculated to the same genus, species, or strain designation.

*Advantages of Approach.* Characterizing biodiversity and subsequently monitoring it with new sequence detection technologies require a complimentary approach that includes both “narrow and deep” sequence interpretation paired with “broad and shallow” sentinel marker scanning for biological warfare agent (BWA) identification. This two-pronged strategy is critical for a comprehensive biothreat assessment program, given the diversity of threat agents that exist and is capable of monitoring both broadly across taxa and deep within a genome. Probabilistic approaches allow fast processing of large amounts of data and provide statistical confidence to the final product: positive identification of bioagents based on signature distributions and patterns [56]. The approach is not dependent on genome assembly nor contiguous sequence nor on full nucleic acid extraction.

The high-throughput nature of sequencing devices inevitably produces sequencing errors to some extent. The errors in standard genome sequencing projects can be readily reduced, if recognized. Potential sequencing errors can also be further minimized by posterior computational processes [57, 58]. A computational method for differentiating target sequences from background sequences and assembling the chosen target sequences is required. A similar, albeit much simpler task has been successfully applied by Salzberg et al. [59, 60] in which endosymbiotic bacterial genomes were readily assembled from excess insect host genome sequences. Technologies involved in assembling microbial genomes from mixed community have been reviewed [61].

Dramatic advances in DNA sequencing and sequence analysis in the last decade have opened up vast amounts of genetic evidence for forensic scientists to investigate. Because of new insights into biodiversity and sample biocomplexity, new ways to process and interpret sequence data is required. Several challenges to use volumes of sequence data have limited analysis to mapping sequence reads and to assemble genomes. New tools and pipelines are needed to address gaps and validate direct sequence analysis of populations. The first gap is a need for improved methods to validate sample data analysis in a nonarbitrary fashion. For this challenge, a calibrant for direct sequence analysis of populations was developed. Second, is a process to compare sample genomes without complete dependence on reference databases. Considering the practically limitless number of microbes in the world and their ever evolving genetic composition, no reference database can ever be sufficient to nonarbitrarily characterize ancestral relationships between samples. In circumstances where fractions of genomic information are used, probabilistic scoring is required to achieve accurate genome identification and a pipeline that accounts for system errors.

Hence analysis of the population, rather than a single target, enables broad characterization of the sample and deep resolution to pinpoint the association between materials and source. Population-Sequencing was performed in blinded fashion and genome identification was based on probabilistic values for matching criteria.

Microbial forensics needs a paradigm shift away from the clonal isolate versus reference genome approach to a comprehensive analysis of genetic content. Foremost, real world samples rarely exist as homogeneous populations of identical clones. Even populations that derive from single cells/viruses acquire mutations that are not equally shared by all members. Metagenomic populations in samples require bioinformatics approaches that deviate away from the clonal isolate paradigm to accommodate potentially hundreds of distinct genomes that may or may not be part of any database. The challenge for bacterial forensics is how to account for the diversity of sample material from a reference. Traditionally, this has been interpreted as how to compare the genetic variation of a single target out of many from a mixture. Comprehensive characterization of the populations would improve the confidence of matching sample to source and define the range of variation from a composite, but finite genetic content of source. As material is manipulated and the ancestry of the material to source increases, variation will become part of the population, but the extent of variation will be constrained by the diversity within the source. Hence the future of bioforensics is in population comparison, namely, Population-Sequencing.

### 4.3. Population-Sequencing

Not only are specific threats of interest identified, without prior knowledge of the sample, but contaminants, related strains, and other genomes of the population are detected, their variation cataloged and probabilities for confident genome identification are indicated. These advantages would potentially be used for attribution, for example, linking materials to their source or to track theft of bacterial strains or manipulation of strain or ones that have been engineered. It also could be used to enhance the security of bacteria being studied in labs and discourage misuse of biological pathogens.

Sequence reads are matched according to stringent bioinformatics criteria and based on the genetic relatedness of neighbor microorganisms. Related organisms share portions of their core genomes, which is an advantage for Population-Sequencing approach because it builds the statistics of assignment to the correct genus and species. Resolution down to the strain level is possible because many pan-genome fragments unique to that strain are also detected. This is referred to as the “cloud” of genetic relationships. The definition of genus or species or other taxonomic categories is made in relation to the core and pangenomes. The diversity of genetic content that defines the species can be considered to be a cloud of possibilities, hence cloud sequencing.

Bioinformatics tools, like Population-Sequencing approaches, handle deep sequencing and population wide comparison for accurate population identification. Bioinformatics tools need to be evaluated to measure genetic change of populations to assess (1) accurate identification of sequence of biothreats, (2) capability to compare genetic content in samples, (3) measurement of relatedness between samples, and (4) calculated distance based on sequence information and finally to establish sound interpretation of comparisons, indicating the strength of the link between evidence and potential sources.

Hence a flexible, on-the-fly, system is needed to measure the comprehensive spread of genomes in samples, rather than looking for one or two key threat agents. Comprehensive analysis of sample populations is required to obtain broad and narrow characterization: broad across the populations representative of different genus, species, and so forth, and narrow down the phylogenetic tree to pinpoint the strain and SNP variants thereof. The aspect of confidence also needs to be considered, ignored by PCR and provided that there are no degeneracies, amplifies a single target. However, there will be endless design and redesign of PCR assay as sequence space knowledge increases every week.

### 4.4. Population-Sequencing Utility for Forensics

The sample itself becomes the database for comparison. Comprehensive analysis of the genetic constituents establishes the sample profile. Profiles can be compared to determine the relatedness of the populations in the samples, not just one or two selected targets. Comprehensive analysis also strengthens the probability of the match and increases the confidence to include and exclude samples as matches.

Assuming that the background detection is real variants, single colonies produce bacterial populations that persist and proliferate with growth/time. Direct sequencing was applied and sequence data was assembled via classical sequencing techniques and evaluated with novel alignment-free bioinformatics tools. Confidence thresholds and probabilistic matching provide bacterial strain name and a confidence score of the identification, in addition to providing the genus and species identification. From the sequence comparison, a sequence profile of the sample can be established. Population-Sequencing transcends the limits of classical sequencing of single clones, because the process avoids genome assembly to cluster and compare sequence information between samples. Sequence information is weighted and strings are assigned probabilistic value. This process is not static, although DNA signatures or other specific sequence information, for example, MLST sites, can be incorporated into the measurements. Profiles of samples can be compared and hence, traceability can be determined, whereby attributing evidence to source. Profiles can be compared to assess relatedness at a genome-scale rather than using single gene phylogenies. This is not based on trying to develop one thousand targets for a PCR assay of a panel of favorite genes and it avoids sample culture. Instead of sequencing clones one-at-a-time, the entire sample is sequenced and genomes identified. This is not about preselection of certain targets, but rather any and all sequence fragments can be used to determine the answer.

While metagenomic analysis enables characterization of the genetic content of the sample and hence the establishment of a sample profile, which can be used to compare other sample profiles to determine attribution, the strains identified may have mutations from the reference. In addition, each sample becomes a unique database of genetic content. Other samples can be compared to that profile, even in the absence of reference sequence databases to name every genome. If the intent is to compare genetic content, then methods that provide a comprehensive description that can be compared to other samples, will establish the approach to distinguish between intentional and natural events, because the directions of genetic variation of sample population genomes will be different. Also comprehensive characterization will provide the boundaries of sample variations, creating quantitative values for sample-to-sample comparisons, to determine when two samples are the same but (slightly) different.

## 5. Summary

This paper outlines salient features of types of artifacts and errors that need to be addressed and challenges for sequence analysis of populations as a method to characterize samples beyond one target microorganism to characterization of populations to achieve comprehensive sample content-multiple genomes, pathogenic and commensal. Data-rich sequence information means that multiple genomes can be identified [57]. However, multiple target analytics tools are needed to improve statistical confidence to trace ancestry of samples and attribute samples to source with probabilistic certainties on many targets instead of a single genome or a single arbitrary target. Population-Sequencing creates a profile of the sample. It interrogates all sequence read data, compares these to sequence fragments, and identifies genomes to which they belong: ranking and scoring major and minor genome constituents of samples. The evidence is now convincing that strain to strain variation in genomic sequence renders probabilistic identification from direct sequence the method of choice for first responders to determine agent identification. The development and testing of selective bioinformatics tools that account for sequencing artifacts and interpret biodiversity are essential for a strong national defense. Direct “Population-Sequencing” has dual benefits for national security and for environmental/clinical health.

Development of a calibrant to normalize across different experiments was also introduced. Collectively, our results demonstrate that the performance of direct sequence analysis of population(s) is acceptable for bacteria strains identification to reference strains and provide useful guidelines for using Population-Sequencing for forensic applications as well as in environmental genome detection with strains other than the reference strain.

## 6. The Way Forward

Microbial forensics recommendations leverage biodiversity and the effects of evolutionary pressures on DNA to focus on innovative and effective approaches to identify, compare, match, and exclude bacterial samples. Recommended research to improve bioforensics includes the following.

- Development of Direct Sequencing techniques for heterogeneous samples.
- Development of algorithms adapted to meet the needs of the forensics community.
- Informatics tools which handle genome-scale data for comparisons.
- Informatics tools which analyze population-based genomic elements and mutation clues from evidence samples routinely ignored by static marker analyses.

At the heart of Population-Sequencing is a revolutionary set of technologies that enable accurate identification and traceability of biological samples/species in a forensic environment was reviewed. Direct and comprehensive genome analysis is dependent on key goals of comparative population analysis, direct sequencing, genome modeling, and database design to create an end-to-end identification system designed for forensic use.

The future of biodetection will likely characterize all microbes in a sample, enabling use of all of the genome regions, which are under different evolutionary pressure, to improve traceability to source. This is important because of gaps in capability of phylogenetic analyses produce different measures of relatedness depending on what single genes are compared. Experimental evidence has shown that direct sequence analysis of environmental samples links sample to source.

- New algorithms based on species biodiversity and sequence signatures.
- Subspecies organism identification in a metagenomic background.
- Fast, accurate probabilistic matching that gives user % confidence of match.
- Filter-in key processes of the culture conditions, for example, the type of medium that may further limit the field of possible biothreat sources.
- Validating “knowledge” pipeline, and moving away from “static” marker molecular signatures, which are limited by erosion.
- Using deep-sequencing to characterize “slow” and “fast” evolved sequence regions.
- Comprehensive analysis of select agent population dynamics and host-environmentpathogen interaction to serve as critical background knowledge.
- Verification that our approach, algorithms/reformatted Database are sufficient.

Because every sample will have a different genetic content (or available pan-genome potential), every sample will be distinguishable from the next. Related samples or samples of common origin will have a probabilistically high similarity of events. Direct Population-Sequencing will further our understanding of community genetics and provide analytical tools to balance abundance of potential knowledge obtained from high-content sequencing to enable insights into the context of the dynamics and relationships of genome populations. Application of Population-Sequencing culture independent methods to clinical samples from patients with a multitude of diseases will lead to an unprecedented rate of detection of pathogens, establishing the link between microorganism populations and human disease and provide comprehensive contextual characterization of emerging and novel agents.
